# A Narrative Review on Stress and Itch: What We Know and What We Would Like to Know

**DOI:** 10.3390/jcm13226854

**Published:** 2024-11-14

**Authors:** Nicole B. Khalil, Giulia Coscarella, Firdaus S. Dhabhar, Gil Yosipovitch

**Affiliations:** 1 Dr. Phillip Frost Department of Dermatology and Cutaneous Surgery, Miami Itch Center, Miller School of Medicine, University of Miami, Miami, FL 33136, USA; nbk23@med.miami.edu; 2 Dermatologia, Dipartimento di Medicina e Chirurgia Traslazionale, Università Cattolica del Sacro Cuore, 00168 Rome, Italy; giuliacoscarella@gmail.com; 3 UOC di Dermatologia, Dipartimento di Scienze Mediche e Chirurgiche, Fondazione Policlinico Universitario A. Gemelli-IRCCS, 00168 Rome, Italy; 4 Department of Psychiatry and Behavioral Sciences, Sylvester Comprehensive Cancer Center, Miller School of Medicine, University of Miami, Miami, FL 33136, USA; fdhabhar@miami.edu; 5 Department of Microbiology and Immunology, Miller School of Medicine, University of Miami, Miami, FL 33136, USA

**Keywords:** itch, stress, mechanism, pathway, inflammation, itch–scratch cycle, treatment

## Abstract

Itch is one of the most prevalent symptoms experienced by patients with inflammatory skin conditions, yet it is also one of the most debilitating. Patients suffering from chronic itch have been found to have significantly higher stress levels compared with those not experiencing itch. In fact, recent studies have revealed a bidirectional relationship between stress and itch, where each condition worsens the other. This is thought to be driven by the vicious itch–scratch cycle, which is fueled by underlying inflammation. The precise molecular pathways and mediators involved, however, remain unclear. This narrative review discusses the existing research on the relationship between stress and itch and outlines future research directions that will be necessary to advance our understanding and treatment of these conditions. Given that the effective management of both symptoms often requires a combined treatment approach, further investigation into their shared mechanisms is essential for identifying successful therapies and improving patient outcomes.

## 1. Introduction

Pruritus is a common manifesting symptom of numerous medical and psychological diseases. Chronic pruritus (CP) is defined as pruritus that lasts for six weeks or more, and it has been shown to lead to a significant decrease in quality of life, comparable to that of chronic pain, making it one of the most debilitating symptoms a patient could experience [1,2].

Pruritus leads to over 7 million ambulatory visits annually in the US. Among those, nearly one-third are due to CP. This figure excludes conditions classically known to cause itch, like atopic dermatitis (AD), thus actually underestimating the overall burden of itch on patients [3]. The prevalence of itch in adults worldwide is 39.8%, with CP specifically affecting between 8% and 25.5% of people [1,4,5]. Although it affects all age groups, CP is most common in the elderly, with a prevalence of 43.3% in this population specifically. Additionally, African American and Asian patients seek care for pruritus more often than other ethnic groups [1].

Despite being primarily considered a consequence of dermatological conditions, CP can also stem from systemic, neurological, psychiatric, and gynecological diseases. Beyond organic causes, psychogenic causes, such as stress, remain prevalent among patients suffering from CP [1]. Indeed, CP and chronic stress can be considered two sides of the same coin. Pruritus can exacerbate mental health conditions, contributing to worse psychiatric outcomes and harder-to-manage disease. Likewise, chronic stress, as well as depression and anxiety, can exacerbate pruritus by intensifying itch perception [6]. In fact, CP is notably common among psychiatric patients, yet it is frequently underreported, making comprehensive patient screening critical to improving treatment outcomes [7]. Large cohort studies have demonstrated a linear relationship between pruritic symptoms and psychological stress, with increasing stress levels being associated with worse pruritus perception [8].

This narrative review aims to determine the relationship between stress and CP as well as to explore the simultaneous therapeutic management of these conditions.

## 2. Methods

A review of the literature was performed using the PubMed and Google Scholar databases. The search keywords included “Chronic stress” OR “Acute stress” OR “Psyche” AND “Chronic pruritus” OR “Itch”. The pertinent literature was compiled for use in this narrative review.

## 3. Stress: Definition and Pathophysiology

Stress is a term that is often used in society to describe the pressures of everyday life [9]. Stress has been defined as a sequence of events that occur when a stimulus (stressor) triggers a reaction in the brain (stress perception), thus resulting in the activation of the body’s fight-or-flight system (biological stress response) [10,11]. The neurotransmitters epinephrine and norepinephrine are released by the sympathetic nervous system in order to mediate the body’s response to stress. Other major mediators of the stress response include corticotropin-releasing hormone (CRH), adrenocorticotropin (ACTH), and cortisol, all of which are produced upon activation of the hypothalamic–pituitary–adrenal (HPA) axis. Receptors for one or more of these mediators are present on nearly every cell in the body, making the effect of stress very powerful and widespread [9,10].

Stress can be further categorized as acute or chronic based on the duration of the stress response. Acute or short-term stress refers to stress lasting from minutes to hours, whereas chronic stress describes stress lasting for days, months, or even years. The stress response produced by the body under conditions of acute stress has been crucial in the survival and reproduction of nearly all living species [11]. Additionally, acute stress has been shown to enhance immune responses and post-surgical healing in humans. Chronic stress, on the other hand, has detrimental effects on the health and wellbeing of humans, primarily through maladaptive modulation of the immune system and hormonal signaling. The same key players are active in both acute and chronic stress, but prolonged exposure to these chemical mediators results in the remodeling of signaling pathways and organ systems in ways that produce deleterious effects on health [12].

The human stress response relies primarily on the structures comprising the HPA axis, as well as the locus coeruleus (responsible for arousal), sympathetic nervous system (SNS), sympathetic-adrenal medullary (SAM) system, parasympathetic nervous system (PNS), mesolimbic and mesocortical dopaminergic systems (responsible for reward and motivation), amygdala (involved in fear perception), and central circadian rhythm system. Under stress, the SNS, SAM system and HPA axis are activated, resulting in the release of norepinephrine, epinephrine, CRH, and vasopressin. CRH and vasopressin stimulate ACTH release from the anterior pituitary gland, which in turn stimulates the adrenal glands to produce and secrete glucocorticoids, namely cortisol [13]. These molecules work to induce physiological changes that are beneficial when faced with a threat. In order to maintain homeostasis, the body has developed feedback loops and regulatory pathways that work to terminate the stress response. Disruption of these pathways under conditions of chronic stress leads to deleterious effects, such as immune suppression and inflammation [10,14].

Chronic stress exposure induces an immune response that leads to the release of inflammatory markers such as cytokines, prostaglandins, and chemokines. These substances affect the HPA axis, promoting the release of glucocorticoids and altering neurotransmitter production and signaling [15,16]. Repetitive activation of the HPA axis results in increased susceptibility to disease and infection, decreased response to vaccines, and impaired wound healing [10,11,17].

Stress is also known to modulate the immune system in a way that increases systemic inflammation, worsening pruritic skin conditions. In fact, stress and itch have been shown to interact with one another through a bidirectional loop driven by the itch–scratch cycle. Numerous studies have demonstrated increased itch in people under increased stress levels [18,19,20,21,22]. Furthermore, a linear relationship between stress levels and itch severity has been revealed, suggesting a direct correlation between the two [18,22]. This phenomenon is believed to be the result of dysregulation of the HPA axis, SNS, and PNS, as well as the activation of a cutaneous neuroendocrine axis. CRH, produced by keratinocytes, mast cells, and sebocytes, binds to the CRH receptor, stimulating the production of several pruritogenic compounds, including substance P (SP), nerve growth factor (NGF), histamine, and acetylcholine (Ach). SP, Ach, and CRH can further activate mast cells, releasing proinflammatory cytokines such as IFN-γ, TNF-α, IL-1, IL-2, IL-4, IL-6, IL-13, and IL-31 [14,23,24]. Among these, IL-4, IL-13, and IL-31 are particularly involved in itch perception. Moreover, chronic stress has been shown to reduce CD4+ and CD8+ T cell levels, shifting the T cell response from protective to suppressive [10]. Environmental stressors and mechanical stress on the skin can stimulate keratinocytes to produce thymic stromal lymphopoietin (TSLP), a key factor in the pathogenesis of AD. TSLP directly promotes the Th2 differentiation of naive T cells, leading to increased production of IL-4 and IL-13, which are key mediators of itch [24]. Table 1 provides an overview of the differences that this article will explore between acute and chronic stress as they relate to itch.

## 4. Acute Stress and Itch

Research on the effect of acute stress on itch has shown inconsistent findings. A study conducted in 2019 by Mochizuki et al. compared acute nonhistaminergic itch combined with acute psychological stress in atopic dermatitis patients versus healthy controls. The results showed that overall itch intensity was reduced following exposure to acute stress stimuli in AD patients. However, there was also an increase in off-site scratching behavior, highlighting a separation between itch perception and patient response [25]. This discrepancy may be due to stress-related inhibitory pathways during social stress. Interestingly, stress has not been directly linked to itch induction but rather to increased scratching and the consequent itch sensation, as demonstrated by AD patients [26,27]. The persistent scratching caused by repetitive acute stressful events can worsen itch, inducing the “itch scratch cycle”, explaining why treatments may not fully work.

In 2010, a study was conducted on AD patients to further investigate the autonomic nervous system’s response to itch, scratching, and mental stress. The results revealed that acute stress caused abnormal heart rate variability, indicating impaired autonomic neural function, particularly marked by sympathetic overactivation. Furthermore, that study observed an elevated baseline of parasympathetic activity, along with a diminished parasympathetic response [28].

To better understand the effect of acute stressors on itch, an experiment was conducted on rats who were exposed to strong, intermediate, and weak levels of stress antinociception (SIA). Those findings showed that itch-related responses decreased under strong SIA, suggesting that acute stress exerts an inhibitory effect on the itch-signaling pathway. Conversely, intermediate and weaker levels of SIA had weaker antipruritic effects compared to higher stress levels [27].

Corticotrophin releasing factor (CRF) is believed to play a role in suppressing stress-induced atopic dermatitis and its associated symptoms [29]. Current evidence emphasizes that acute stress triggers defensive responses through the activation of the sympathetic adrenal (SA), HPA, and non-noxious afferent (NNA) pathways, as well as through the release of proinflammatory mediators to restore homeostasis. However, intense, acute stress can lead to heightened neuroimmune system activation, resulting in clinical rashes, edema, pruritus, or pain [30,31,32,33].

For example, increased oxidative stress and redox impairment due to acute psychosocial stress can affect proinflammatory cytokine levels, as observed in students preparing for exams.

Acute psychological stress, along with sleep deprivation and nutritional factors, impacts the integrity of the epidermal barrier, immune response, and neurogenic factors, worsening inflammatory diseases and pruritus [34,35,36]. Sleep loss in particular causes elevated cortisol levels, impairing maintenance of the skin barrier, thus aggravating skin dryness and promoting the consequent itch sensation [35].

Research in this area is limited, prompting the need for further exploration of the interactions between acute stress and itch.

## 5. Chronic Stress and Itch

Unlike acute stress, chronic stress is known to negatively affect patients with itchy skin conditions. Chronic stress activates the HPA axis and the SNS, resulting in elevated levels of norepinephrine, cortisol, and other stress mediators, as well as changes in the receptor sensitivities for these molecules [23]. These alterations lead to increased activation of keratinocytes and mast cells, which produce pruritogenic molecules like NGF, histamine, Ach, and numerous cytokines, discussed above. Ach, for example, increases itch sensation frequency by stimulating pruritogenic receptors on unmyelinated C fibers in the skin and promoting histamine release from mast cells. NGF, on the other hand, encourages the maturation of peripheral sensory and sympathetic fibers, lowering the itch threshold and making it easier for patients to experience itch [23].

A study conducted on mice exposed to a four-week water-avoidance stress test showed a tendency to develop intense scratching and dermatitis after stress exposure [29]. Consistent with these findings, mice with allergic contact dermatitis subjected to the stress of chronic social isolation also exhibited increased scratching behavior in areas distinct from the dermatitis site following stress exposure [37]. Furthermore, a 10 day water-avoidance stress test was conducted on mice that demonstrated augmented scratching after the injection of compound 48/80, a mast cell degranulator [38]. Another animal study, using a 9-day heterotypic chronic intermittent stress protocol, led to increased scratching after serotonin injection in rats [39]. Additionally, a four-week chronic unpredictable mild stress protocol also resulted in increased scratching following histamine or chloroquine injections, as well as an increase in spontaneous scratching in allergic contact dermatitis model mice [40].

Similar results have been reported in human studies as well. A large cohort study in Japan, for example, compared the frequency of itch reported by patients to their perceived stress levels over a one-month long period. Those results showed not only that patients with pruritus had higher levels of stress than those without pruritus, but also that there is a linear relationship between the two, with increasing stress levels correlating to increased itch frequency [8]. Furthermore, a large survey-based cross-sectional study performed in the US also demonstrated a significant correlation between stress and itch in undergraduate students [18]. Similar cross-sectional studies conducted in Australia on undergraduate students and on Saudi Arabian medical students revealed a significant correlation between increased levels of chronic stress and itch [19,20]. Similarly, a recent large survey-based study conducted in Germany showed that highly stressed students experienced itch significantly more frequently than students experiencing low stress [21].

## 6. Understanding the Bidirectional Relationship Between Chronic Pruritus and Stress

Chronic stress has been identified as a significant trigger for pruritus, though the exact pathogenetic mechanism remains unclear. Psychiatric and psychological impairments are likely to cause CP as a physical manifestation of mental illness [41,42,43,44]. Specifically, the ICD-10 classification system refers to CP of psychological origin as “somatoform pruritus”, which is believed to result from neurotic conflicts or structural deficits in the psychiatric system.

Stress is well-known to influence both itchy skin conditions and healthy skin. An investigative analysis revealed that significant stressful life events were linked to cutaneous sensory manifestations, with pruritus being the most common symptom experienced by patients [45].

Several studies have already been conducted on the potential link between stress, immune system alterations, and changes in the inflammatory system [46,47]. Psychological stress has been shown to be involved in comorbidities in which immune dysregulation is implicated in the pathogenesis. This may be due to the increase in proinflammatory cytokines and acute-phase protein levels consequent to stress and anxiety disorders [48].

Low-grade inflammation is emerging as a potential cause of pruritus sensation in response to stress. The increased HPA axis activity due to stress results in an inflammatory response that leads to a change in sensitivity of the adrenergic and glucocorticoid receptors on the immune cells [49,50].

Consequently, chronic stress induces remodeling of the forebrain, thus resulting in dysfunctional vagal brake and loss of parasympathetic activation. Most of the diseases caused by parasympathetic impairment—for example, heart failure—are predominantly accompanied by pruritus [23].

In 2020, a study explored the association between perceived stress and hypothalamic volume in patients with AD by collecting brain anatomy images [51]. That study revealed a negative correlation between perceived stress levels and the gray-matter volume of the hypothalamus, specifically in the paraventricular nucleus (PVN). Since the PVN is crucial in regulating the HPA axis and sympathetic-adrenal medullary (SAM) activity, it is reasonable to hypothesize that their dysregulation due to chronic stress could lead to hypothalamic volume reduction. Furthermore, that study suggests that the frequency of stressful events throughout the lifetime may influence the hypothalamic volume in AD patients [50].

**Figure 1:**
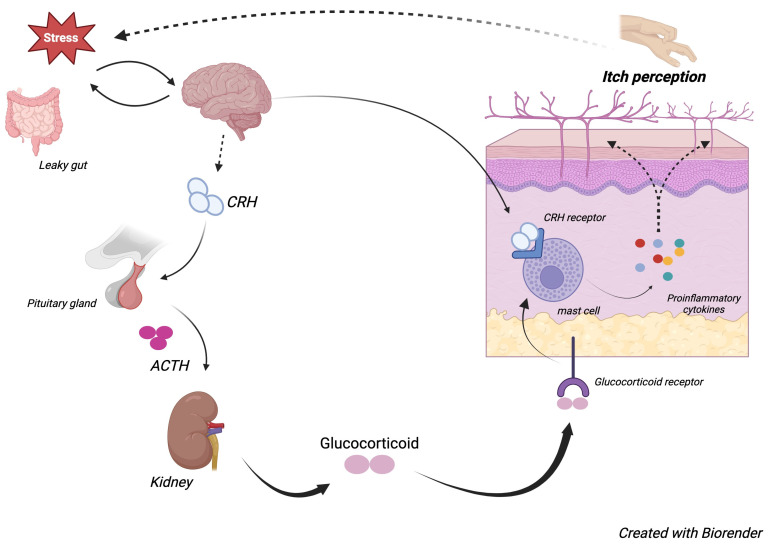
The bidirectional relationship between chronic stress and itch is mediated by the HPA axis, resulting in the release of proinflammatory cytokines in the skin, which increases itch perception and further perpetuates this chronic cycle.

Chronic stress is known to negatively affect the integrity of the skin barrier, disrupting permeability homeostasis and weakening antimicrobial defenses. This, in turn, promotes the release of proinflammatory cytokines to trigger skin-repair mechanisms, further exacerbating itching [52,53,54]. While acute stress primarily triggers the release of proinflammatory type 1 cytokines, the skin responds to chronic stress by coordinating with the HPA axis, leading to the production of CRF by keratinocytes. In fact, upon chronic stress exposure, a separate prolonged HPA axis activation occurs in the skin, leading to the release of chemical mediators from mast cells and keratinocytes, which in turn promotes pruritus signaling, either by activating pruriceptors on nerve endings or releasing pruritogenic substances. Cutaneous CRF promotes the release of proinflammatory cytokines, namely IL-1, IL-6, and TNF-a. In addition to keratinocytes, mast cells, which are involved in neuroimmune interactions in the skin, can release IL-1 during stress and also respond to its stimulation [55,56]. When activated by IL-1, the mast cells secrete various inflammatory cytokines that can shift the immune response balance toward either T-helper (Th)1 or Th2 cells, depending on the nature of the stress exposure. Interestingly, chronic stress-induced fluctuations in catecholamine and glucocorticoid levels have resulted in increases in the numbers of Th2 and cytotoxic lymphocytes, thereby enhancing humoral immunity and suppressing cellular immunity [55,56,57,58]. Other Th2 cytokines that are upregulated under chronic stress include IL-4, IL-10, and IL-13. Neuropeptides are also involved in the perception of itch through their activation of mast cells, with examples including substance P, PAR-2, and CGRP, all of which have been suggested as key mediators of the stress-induced itch pathway [59]. Activated mast cells also release numerous other proinflammatory mediators, such as leukotrienes, prostaglandins, and histamine, further contributing to stress-induced itch (Figure 1) [59].

In addition, chronic stress causes an imbalance in mu opioid and kappa opioid peptides and their receptors in the skin, combined with aberrant activity of the parasympathetic nervous system [51,60]. The parasympathetic nerves, as well as keratinocytes, are responsible for Acetylcholine (Ach) production, which in turn controls histamine release from mast cells [61].

A key mechanism that may explain the elevated levels of proinflammatory cytokines in stress conditions involves the gut–brain axis. In normal conditions, the intestinal epithelial cells form tight connections that prevent harmful substances from entering the bloodstream. However, psychological stress weakens this barrier, leading to stress-related leaky gut (Figure 1). This allows bacteria, viruses, and toxins to enter the bloodstream through the gut, triggering a systemic increase in proinflammatory cytokines. Notably, chronic gastrointestinal diseases caused by leaky gut often occur alongside inflammatory skin diseases, with improvements in gastrointestinal health frequently leading to skin improvement. This connection has been observed in patients affected by inflammatory skin conditions like AD, where leaky gut can be considered the leading mechanism behind stress-induced itch [62].

## 7. Therapeutic Management and Future Perspective

Consequent to the significant correlation between the psychological and dermatological causes of chronic pruritus, an adequate treatment necessitates the combined treatment of both conditions, which could be helpful in coping with the vicious itch–scratch cycle [63].

When combined with pharmacological treatments for itch, education, emotional support, and behavior therapies, such as awareness and relaxation training, can further enhance a patient’s ability to manage and break the itch–scratch cycle. Furthermore, gaining a deeper understanding of triggering factors, along with increased awareness of the disease and the use of itch-relieving interventions, may help to improve patients’ insight and perception, which is crucial when initiating psychological therapies. A meta-analysis that investigated the effects of psychological interventions on itch evaluated their larger effects on symptom severity compared to the extent of the dermatological disease. That study concluded that psychological interventions benefit patients with skin conditions by reducing the severity of their conditions, improving psychosocial outcomes, and decreasing itch/scratch behaviors [64].

Relaxation techniques can also be effective in managing chronic pruritus, particularly when stressful situations and disease flare-ups are linked. Proven methods for reducing itch include progressive muscle relaxation (PMR) and autogenic training (AT). For individuals with compulsive scratching behaviors, such as those affected by prurigo nodularis, habit reversal training (HRT) may be an appropriate initial treatment. HRT aims to replace harmful behaviors with neutral ones by teaching patients to recognize dysfunctional actions and replace them with a competing response. Cognitive behavioral therapies (CBTs), which combine multiple psychological interventions, allow patients to select the approach that best fits their needs, with the goal of restructuring patients’ cognition. CBT has been successful in reducing chronic itch in adult patients with AD. In addition, psychological interventions such as Acceptance and Commitment Therapy (ACT) and Mindfulness-Based Stress Reduction (MBSR), which are already known for their effectiveness in the management of chronic pain, may be effective in treating chronic pruritus [65].

After having assessed the complex interplay between chronic stress and chronic pruritus, psychological interventions should be considered valuable supplementary treatment for patients with CP. Integrating both medical and psychological therapies would likely yield positive outcomes for patients, as stress and itch exacerbate each other. It is therefore important to utilize interventions that reduce stress, including pharmacological treatments and integrative medicine, to treat stress-induced itch.

## 8. What We Would Like to Know

In addition to the aforementioned need for exploring combined treatment modalities, further research is required to understand the exact mechanism underlying the relationship between stress and itch and, in particular, the key players involved. The prevailing hypothesis suggests that dysregulation of the HPA axis via dysregulated activation of the sympathetic and parasympathetic nervous systems is the foundational process linking stress and itch. However, the exact molecular interactions remain unclear.

Neuronal tracing studies have intriguingly revealed that the neurons in the parabrachial nucleus project axons to the central amygdala (CeA). Additionally, the amygdala has been shown to activate in response to pruritic stimuli [66,67]. Under chronic stress, hyperexcitability of the lateral nuclei of the amygdala (LA) occurs, increasing stress and anxiety levels, which are known to correlate to the itch sensation [67,68]. Plausibly, chronic stress could therefore enhance itch, at least partially, through this amygdala-dependent pathway.

The role of inflammation in the pathogenesis of CP has been well-established. This therefore leads us to hypothesize that therapies targeting the inflammatory reactions and mediators that promote itch could be used in the treatment of CP. Recent studies have started to investigate the potential of vagus nerve stimulation (VNS) in the treatment of numerous diseases characterized by inflammation, as well as in stress-induced psychiatric disorders specifically [69]. Afferent vagal nerve fibers transmit sensory information to the brain regions involved in the stress response, including the amygdala, hippocampus, insula, and anterior cingulate/prefrontal cortex, making them potential targets in the treatment of diseases promoted by stress, such as CP. Preclinical studies have shown promising results, demonstrating that left cervical VNS can reduce inflammation by activating the efferent arm of the inflammatory reflex. This promotes the production of anti-inflammatory Ach, which counteracts the proinflammatory state by suppressing the production of numerous inflammatory cytokines [70]. A study testing noninvasive VNS in patients with PTSD yielded significant results, suggesting that this therapy could effectively reduce stress levels and improve mental health [69]. Consequently, there is reason to believe that further research may prove VNS to be effective in treating stress-induced CP.

Moreover, recent research on the role of orexin neurons in the perception of itch raises the question of whether these could also be involved in the pathway underlying stress and itch. Orexin-producing neurons in the hypothalamus, also called Hypocretin-1 and Hypocretin-2, are neuropeptides derived from pre-pro-orexin under numerous physiological states, including arousal and stress processing, to induce a fight-or-flight response. They are considered to be a master switch controlling itch and pain perception and are activated by pruritic stimulation. When activated, orexin neurons inhibit pain and therefore facilitate itch, as the two are antagonistically regulated. Scratching the skin to remove irritants is an example of a fight-or-flight response developed to enhance survival, which occurs due to orexin neuron activation [71]. This demonstrates the mediation of stress and itch by orexin at a local level. However, whether orexin is involved on a more systemic level remains to be determined.

Regarding the molecular interactions taking place at a cellular level, the intricate interplay between cytokine imbalance and chronic stress has been extensively studied in recent years, revealing profound implications for understanding and addressing various health conditions. Cytokine upregulation has been consistently linked to neurotransmitter imbalances in the brain and behavioral changes such as depression in both animals and humans, underscoring the critical role of cytokine imbalance in the pathogenesis of stress-related disorders. In fact, psychotropic drugs that modulate neurotransmitter levels, such as selective serotonin reuptake inhibitors (SSRIs), tricyclic antidepressants (TCAs), benzodiazepines, and GABA analogs, have been shown to effectively treat both mood disorders and itch. However, whether they alleviate itch and mood symptoms through a common pathway or separate pathways remains unclear [72]. When exposed to antigens or external stimuli, keratinocytes become activated and release cytokines and chemokines essential for regulating inflammatory and immune responses within the skin through the activation of antigen-presenting cells (APCs) [32,51,73]. Under psychological stress, keratinocytes, along with mast cells, significantly increase cytokine secretion. The role of Th2 cytokines in itch has been well-established. However, their role in stress is unclear. Furthermore, studies suggest that the Th1 cytokine TNF-a may also be implicated in this process, as treatment with anti-TNF-a agents has led to improvement in both itch and mood symptoms, including stress, especially in psoriasis [72]. The roles of Th17 and IL-23 in stress-related itch remain to be elucidated.

The isolation of precise pathways and molecular mediators/modulators of the pathways shared by stress and itch will be critical for the development of novel therapies as well as for understanding why current therapies work.

## 9. Conclusions

CP involves complex interactions between the nervous, immune, and psychological systems, with stress playing a significant role in exacerbating symptoms. Effective management requires a combined approach of medical treatments and psychological interventions. Further research into the shared mechanisms between CP and chronic stress could aid in developing unified therapeutic approaches that enhance patient outcomes and quality of life by simultaneously interrupting the vicious cycle of itch and stress.

## Figures and Tables

**Table 1:** Brief overview of the major differences between acute and chronic stress as they relate to the pathophysiology of itch.

	Effect on Skin Health	Findings in Experimental Models	Impact on Itch-Scratch Cycle	Role of CRF	Immune and Inflammatory Responses	Effects on Itch
Acute Stress	Temporarily enhances wound healing but may promote rashes, edema, or pain	Results in separation of itch perception and response	Does not directly induce itch; Increases scratching, resulting in secondary itch	CRF inhibits HPA axis activation, decreasing itch	Temporary release of inflammatory markers strengthens immunity	High levels of acute stress may suppress itch; lower levels have weaker effects
Chronic Stress	Weakens skin barrier and promotes inflammatory dermatoses	Levels of chronic stress have a linear relationship with itch frequency	Encourages persistent scratching, leading to chronic itch and disease progression	Sustained release of CRF disrupts HPA axis and normal homeostatic feedback loops	Consistently heightened inflammatory markers leads to impaired immunity	Lowers the itch sensation threshold and disrupts HPA axis, resulting in increased itch

## References

[1] Roh Y.S., Choi J., Sutaria N., Kwatra S.G. (2022). Itch: Epidemiology, clinical presentation, and diagnostic workup. J. Am. Acad. Dermatol..

[2] Ständer S., Grundmann S.A. (2012). Chronic pruritus. G. Ital. Dermatol. Venereol. Organo Uff. Soc. Ital. Dermatol. Sifilogr..

[3] Shive M., Linos E., Berger T., Wehner M., Chren M.M. (2013). Itch as a patient-reported symptom in ambulatory care visits in the United States. J. Am. Acad. Dermatol..

[4] Yosipovitch G., Yosipovitch G., Skayem C., Aroman M.S., Taieb C., Inane M., Hayoun Y.B., Cullel N.P., Baissac C., Halioua B. (2024). International study on prevalence of itch: Examining the role of itch as a major global public health problem. Br. J. Dermatol..

[5] Hay R.J., Johns N.E., Williams H.C., Bolliger I.W., Dellavalle R.P., Margolis D.J., Marks R., Naldi L., Weinstock M.A., Wulf S.K. (2014). The global burden of skin disease in 2010: An analysis of the prevalence and impact of skin conditions. J. Investig. Dermatol..

[6] Ju T., Yosipovitch G. (2020). Neuropathic pruritus associated with brain disorders. Itch.

[7] Dalgard F.J., Svensson Å., Halvorsen J.A., Gieler U., Schut C., Tomas-Aragones L., Lien L., Poot F., Jemec G.B.E., Misery L. (2020). Itch and Mental Health in Dermatological Patients across Europe: A Cross-Sectional Study in 13 Countries. J. Investig. Dermatol..

[8] Yamamoto Y., Yamazaki S., Hayashino Y., Takahashi O., Tokuda Y., Shimbo T., Fukui T., Hinohara S., Miyachi Y., Fukuhara S. (2009). Association between frequency of pruritic symptoms and perceived psychological stress: A Japanese population-based study. Arch. Dermatol..

[9] Senanayake G.B., Arambepola C. (2019). Understanding chronic stress: A narrative review of literature. J. Coll. Community Physicians Sri Lanka.

[10] Dhabhar F.S. (2013). Psychological stress and immunoprotection versus immunopathology in the skin. Clin. Dermatol..

[11] Dhabhar F.S. (2018). The short-term stress response—Mother nature’s mechanism for enhancing protection and performance under conditions of threat, challenge, and opportunity. Front. Neuroendocrinol..

[12] McEwen B.S. (2006). Protective and damaging effects of stress mediators: Central role of the brain. Dialogues Clin. Neurosci..

[13] Agorastos A., Chrousos G.P. (2022). The neuroendocrinology of stress: The stress-related continuum of chronic disease development. Mol. Psychiatry.

[14] Tsigos C., Kyrou I., Kassi E., Chrousos G.P., Feingold K.R., Anawalt B., Blackman M.R., Boyce A., Chrousos G., Corpas E., de Herder W.W., Dhatariya K., Dungan K., Hofland J. (2020). Stress: Endocrine Physiology and Pathophysiology. Endotext.

[15] Nemeroff C.B. (2003). The role of GABA in the pathophysiology and treatment of anxiety disorders. Psychopharmacol. Bull..

[16] Hou R., Baldwin D.S. (2012). A neuroimmunological perspective on anxiety disorders. Hum. Psychopharmacol..

[17] Glaser R., Kiecolt-Glaser J.K. (2005). Stress-induced immune dysfunction: Implications for health. Nat. Rev. Immunol..

[18] Schut C., Mollanazar N.K., Sethi M., Nattkemper L.A., Valdes-Rodriguez R., Lovell M.M., Calzaferri G.L., Yosipovitch G. (2016). Psychological Stress and Skin Symptoms in College Students: Results of a Cross-sectional Web-based Questionnaire Study. Acta Derm.-Venereol..

[19] Stewart T.J., Schut C., Whitfeld M., Yosipovitch G. (2018). Cross-sectional study of psychological stress and skin symptoms in Australian university students. Australas. J. Dermatol..

[20] Bin Saif G.A., Alotaibi H.M., Alzolibani A.A., Almodihesh N.A., Albraidi H.F., Alotaibi N.M., Yosipovitch G. (2018). Association of psychological stress with skin symptoms among medical students. Saudi Med. J..

[21] Kiupel S., Kupfer J., Kottlors S., Gieler U., Yosipovitch G., Schut C. (2023). Is stress related to itch in German students? Results of an online survey. Front. Med..

[22] Halvorsen J.A., Dalgard F., Thoresen M., Thoresen M., Bjertness E., Lien L. (2009). Itch and mental distress: A cross-sectional study among late adolescents. Acta Derm.-Venereol..

[23] Golpanian R.S., Kim H.S., Yosipovitch G. (2020). Effects of stress on itch. Clin. Ther..

[24] Cevikbas F., Lerner E.A. (2020). Physiology and Pathophysiology of Itch. Physiol. Rev..

[25] Mochizuki H., Lavery M.J., Nattkemper L.A., Albornoz C., Valdes Rodriguez R., Stull C., Weaver L., Hamsher J., Sanders K.M., Chan Y.H. (2019). Impact of acute stress on itch sensation and scratching behaviour in patients with atopic dermatitis and healthy controls. Br. J. Dermatol..

[26] Ständer S. (2019). How acute stress impacts the itch-scratch cycle in atopic dermatitis: A clinical lesson. Br. J. Dermatol..

[27] Spradley J.M., Davoodi A., Carstens M.I., Carstens E. (2012). Effects of acute stressors on itch- and pain-related behaviors in rats. Pain.

[28] Tran B.W., Papoiu A.D., Russoniello C.V., Wang H., Patel T.S., Chan Y.H., Yosipovitch G. (2010). Effect of itch, scratching and mental stress on autonomic nervous system function in atopic dermatitis. Acta Derm.-Venereol..

[29] Amano H., Negishi I., Akiyama H., Ishikawa O. (2008). Psychological stress can trigger atopic dermatitis in NC/Nga mice: An inhibitory effect of corticotropin-releasing factor. Neuropsychopharmacol. Off. Publ. Am. Coll. Neuropsychopharmacol..

[30] Peters E.M. (2016). Stressed skin?—A molecular psychosomatic update on stress-causes and effects in dermatologic diseases. J. Dtsch. Dermatol. Ges./J. Ger. Soc. Dermatol. JDDG.

[31] Herborn K.A., Graves J.L., Jerem P., Evans N.P., Nager R., McCafferty D.J., McKeegan D.E. (2015). Skin temperature reveals the intensity of acute stress. Physiol. Behav..

[32] Pondeljak N., Lugović-Mihić L. (2020). Stress-induced Interaction of Skin Immune Cells, Hormones, and Neurotransmitters. Clin. Ther..

[33] Van Loey N.E., Hofland H.W.C., Vlig M., Vandermeulen E., Rose T., Beelen R.H.J., Ulrich M.M.W. (2018). Associations between traumatic stress symptoms, pain and bio-active components in burn wounds. Psychoneuroendocrinology.

[34] Matalka K.Z. (2003). Neuroendocrine and cytokines-induced responses to minutes, hours, and days of mental stress. Neuro Endocrinol. Lett..

[35] Leproult R., Copinschi G., Buxton O., Van Cauter E. (1997). Sleep loss results in an elevation of cortisol levels the next evening. Sleep.

[36] Korkina L., Pastore S. (2009). The role of redox regulation in the normal physiology and inflammatory diseases of skin. Front. Biosci. (Elite Ed.).

[37] Kitagaki H., Hiyama H., Kitazawa T., Shiohara T. (2014). Psychological stress with long-standing allergic dermatitis causes psychodermatological conditions in mice. J. Investig. Dermatol..

[38] Zhao P., Hiramoto T., Asano Y., Kubo C., Sudo N. (2013). Chronic psychological stress exaggerates the compound 48/80-induced scratching behavior of mice. Pharmacol. Biochem. Behav..

[39] Peng X.Y., Huang Y., Wang X.L., Cao L.F., Chen L.H., Luo W.F., Liu T. (2015). Adrenergic β2-receptor mediates itch hypersensitivity following heterotypic chronic stress in rats. Neuroreport.

[40] Wang X.D., Yang G., Bai Y., Feng Y.P., Li H. (2018). The behavioral study on the interactive aggravation between pruritus and depression. Brain Behav..

[41] Chrostowska-Plak D., Reich A., Szepietowski J.C. (2013). Relationship between itch and psychological status of patients with atopic dermatitis. J. Eur. Acad. Dermatol. Venereol. JEADV.

[42] Reich A., Hrehorów E., Szepietowski J.C. (2010). Pruritus is an important factor negatively influencing the well-being of psoriatic patients. Acta Derm.-Venereol..

[43] Sheehan-Dare R.A., Henderson M.J., Cotterill J.A. (1990). Anxiety and depression in patients with chronic urticaria and generalized pruritus. Br. J. Dermatol..

[44] Stumpf A., Stander S., Warlich B., Fritz F., Bruland P., Pfleiderer B., Heuft G., Schneider G. (2015). Relations between the characteristics and psychological comorbidities of chronic pruritus differ between men and women: Women are more anxious than men. Br. J. Dermatol..

[45] Gupta M.A., Gupta A.K. (2004). Stressful major life events are associated with a higher frequency of cutaneous sensory symptoms: An empirical study of non-clinical subjects. J. Eur. Acad. Dermatol. Venereol. JEADV.

[46] Fleshner M., Crane C.R. (2017). Exosomes, DAMPs and miRNA: Features of Stress Physiology and Immune Homeostasis. Trends Immunol..

[47] Powell N.D., Sloan E.K., Bailey M.T., Arevalo J.M., Miller G.E., Chen E., Kobor M.S., Reader B.F., Sheridan J.F., Cole S.W. (2013). Social stress up-regulates inflammatory gene expression in the leukocyte transcriptome via β-adrenergic induction of myelopoiesis. Proc. Natl. Acad. Sci. USA.

[48] Dimsdale J.E. (2008). Psychological stress and cardiovascular disease. J. Am. Coll. Cardiol..

[49] Juruena M.F., Eror F., Cleare A.J., Young A.H. (2020). The Role of Early Life Stress in HPA Axis and Anxiety. Adv. Exp. Med. Biol..

[50] Slominski A.T., Zmijewski M.A., Skobowiat C., Zbytek B., Slominski R.M., Steketee J.D. (2012). Sensing the environment: Regulation of local and global homeostasis by the skin’s neuroendocrine system. Adv. Anat. Embryol. Cell Biol..

[51] Mochizuki H., Schut C., Shevchenko A., Valdes-Rodriguez R., Nattkemper L.A., Yosipovitch G. (2020). A Negative Association of Hypothalamic Volume and Perceived Stress in Patients with Atopic Dermatitis. Acta Derm.-Venereol..

[52] Slominski A. (2007). A nervous breakdown in the skin: Stress and the epidermal barrier. J. Clin. Investig..

[53] Aberg K.M., Radek K.A., Choi E.H., Kim D.K., Demerjian M., Hupe M., Kerbleski J., Gallo R.L., Ganz T., Mauro T. (2007). Psychological stress downregulates epidermal antimicrobial peptide expression and increases severity of cutaneous infections in mice. J. Clin. Investig..

[54] Berger T.G., Steinhoff M. (2011). Pruritus in elderly patients—Eruptions of senescence. Semin. Cutan. Med. Surg..

[55] Gallenga C.E., Pandolfi F., Caraffa A., Kritas S.K., Ronconi G., Toniato E., Martinotti S., Conti P. (2019). Interleukin-1 family cytokines and mast cells: Activation and inhibition. J. Biol. Regul. Homeost. Agents.

[56] Caraffa A., Gallenga C.E., Kritas S.K., Ronconi G., Conti P. (2019). Impact of mast cells in systemic lupus erythematosus: Can inflammation be inhibited?. J. Biol. Regul. Homeost. Agents.

[57] Conti P., Caraffa A., Mastrangelo F., Tettamanti L., Ronconi G., Frydas I., Kritas S.K., Theoharides T.C. (2018). Critical role of inflammatory mast cell in fibrosis: Potential therapeutic effect of IL-37. Cell Prolif..

[58] Taracanova A., Tsilioni I., Conti P., Norwitz E.R., Leeman S.E., Theoharides T.C. (2018). Substance P and IL-33 administered together stimulate a marked secretion of IL-1β from human mast cells, inhibited by methoxyluteolin. Proc. Natl. Acad. Sci. USA.

[59] Kim H.J., Park J.B., Lee J.H., Kim I.H. (2016). How stress triggers itch: A preliminary study of the mechanism of stress-induced pruritus using fMRI. Int. J. Dermatol..

[60] Bigliardi P.L., Tobin D.J., Gaveriaux-Ruff C., Bigliardi-Qi M. (2009). Opioids and the skin—Where do we stand?. Exp. Dermatol..

[61] Kim H.S., Yosipovitch G. (2013). An aberrant parasympathetic response: A new perspective linking chronic stress and itch. Exp. Dermatol..

[62] Biazus Soares G., Mahmoud O., Yosipovitch G., Mochizuki H. (2024). The mind-skin connection: A narrative review exploring the link between inflammatory skin diseases and psychological stress. J. Eur. Acad. Dermatol. Venereol. JEADV.

[63] Bathe A., Matterne U., Dewald M., Grande T., Weisshaar E. (2009). Educational multidisciplinary training programme for patients with chronic pruritus. Acta Derm.-Venereol..

[64] Lavda A.C., Webb T.L., Thompson A.R. (2012). A meta-analysis of the effectiveness of psychological interventions for adults with skin conditions. Br. J. Dermatol..

[65] Schut C., Mollanazar N.K., Kupfer J., Gieler U., Yosipovitch G. (2016). Psychological Interventions in the Treatment of Chronic Itch. Acta Derm.-Venereol..

[66] Pavlenko D., Akiyama T. (2019). Why does stress aggravate itch? A possible role of the amygdala. Exp. Dermatol..

[67] Sanders K.M., Sakai K., Henry T.D., Hashimoto T., Akiyama T. (2019). A Subpopulation of Amygdala Neurons Mediates the Affective Component of Itch. J. Neurosci. Off. J. Soc. Neurosci..

[68] Rau A.R., Chappell A.M., Butler T.R., Ariwodola O.J., Weiner J.L. (2015). Increased Basolateral Amygdala Pyramidal Cell Excitability May Contribute to the Anxiogenic Phenotype Induced by Chronic Early-Life Stress. J. Neurosci. Off. J. Soc. Neurosci..

[69] Bremner J.D., Gurel N.Z., Wittbrodt M.T., Shandhi M.H., Rapaport M.H., Nye J.A., Pearce B.D., Vaccarino V., Shah A.J., Park J. (2020). Application of Noninvasive Vagal Nerve Stimulation to Stress-Related Psychiatric Disorders. J. Pers. Med..

[70] Falvey A., Metz C.N., Tracey K.J., Pavlov V.A. (2022). Peripheral nerve stimulation and immunity: The expanding opportunities for providing mechanistic insight and therapeutic intervention. Int. Immunol..

[71] Kaneko T., Kuwaki T. (2023). The opposite roles of orexin neurons in pain and itch neural processing. Peptides.

[72] Sanders K.M., Akiyama T. (2018). The vicious cycle of itch and anxiety. Neurosci. Biobehav. Rev..

[73] Bakula A., Lugović-Mihić L., Situm M., Turcin J., Sinković A. (2011). Contact allergy in the mouth: Diversity of clinical presentations and diagnosis of common allergens relevant to dental practice. Acta Clin. Croat..

